# Associations between physical activity and heart disease among middle-aged and older Chinese adults

**DOI:** 10.3389/fcvm.2025.1383888

**Published:** 2025-01-24

**Authors:** Yujia Liu, Chaoqun Yuan, Tong Chen, Linlin Zhou, Gengyin Zhu, Yu Chen

**Affiliations:** ^1^ Institute of Physical Education, Jiangsu Normal University, Xuzhou, China; ^2^ Sports Health College, Chengdu University of Traditional Chinese Medicine, Chengdu, China; ^3^ Library of Jingwen, Jiangsu Normal University, Xuzhou, China; ^4^ College of Physical Education, Chongqing University, Chongqing, China

**Keywords:** physical activity, heart disease, middle-aged and older individuals, dose-response relationship, intensity

## Abstract

**Objective:**

To quantify the risk of heart diseases (HD) and determine the relationship between physical activity (PA) dimensions and HD among Chinese middle-aged and older individuals.

**Methods:**

Using data from the China Health and Retirement Longitudinal Study (CHARLS, 2018), 16,927 participants were included in this study. Multivariate logistic regression was performed to determine the association between HD risk and PA dimensions, including volume, intensity, frequency, and duration. Restricted cubic spline analysis was conducted to assess the dose-response relationship between PA and HD risk.

**Results:**

Compared to the least active participants, a low risk of HD was significantly associated with a higher PA volume. With regards to frequency, a lower HD risk was associated with performing vigorous PA except for 3–5 days/week. The frequency and duration of light as well as moderate PA had no significant associations with HD risk after adjusted by using covariates. A non-linear association was also noted, with increased PA being associated with decreased HD risk, with steeper reductions in HD risk at low activity levels than at high activity levels. There was a non-linear association between PA and HD risk in participants in male and aged older than 65 years.

**Conclusions:**

An inverse non-linear dose-response association was detected between the total volume of PA and HD risk. As PA increased to 4,000 METs-min/week, HD risk in the overall population decreased by approximately 26%, while further increases in PA did not produce any further marked reduction in the risk. A vigorous intensity of PA was associated with a reduced risk of HD and is strongly recommended.

## 1 Introduction

Globally, cardiovascular diseases (CVD) are the leading causes of death. According to the World Health Organization, 17.9 million people die from CVD each year, representing 32% of global deaths (1). It has been postulated that by 2030, this number will increase to ∼23.3 million (1). Heart diseases (HD) include myocardial infarction, coronary heart disease, angina pectoris, and congestive heart failure among others. Myocardial infarction (MI) is associated with 15% of global mortality (2), while heart failure (HF) affects >26 million people globally (3). Cardiovascular disease-associated mortality as well hospitalization rates keep increasing (4). Coupled with increasing health care costs for HD (5), the already overburdened traditional health care model is a cause for concern.

Lifestyle changes are closely associated with fitness (6, 7). Among the least physically active adults, sitting is correlated with all-cause and CVD-associated mortality risk (6), while physical activity (PA) contributes to lowering the risks of CVD in all-age stages (8, 9). PA can reduce the risk of developing MI, moreover, self-reported regular PA is associated with a 45% lower case fatality in MI with a dose-response association between increasing levels of PA and decreased same-day fatality (10). Higher physical activities are associated with low mortality outcomes for coronary heart disease patients (11). PA interventions are effective as therapeutic options for heart failure patients after invasive treatment of angina pectoris (12). However, Bahls et al. denied the causal relationship between PA and sedentary behaviors with the risk of coronary artery disease, myocardial infarction, and ischemic stroke (13). Thus, the bias of the relationship between PA and HD may be influenced by multiple factors.

The characteristics of PA play key roles in HD-associated morbidity and mortality. A higher level of PA has been noted for coronary heart disease (11), while a dose-dependent protective effect of PA on CVD mortality has been reported in older adults (8). Different intensities of PA are recommended to lower HD risk (6, 10). The duration and frequency of PA can also affect the relationship between PA and HD. We aimed at determining the association between PA and the risk of HD morbidity among Chinese middle-aged and older individuals. We used the China Health and Retirement Longitudinal Study (CHARLS) data from 2018, which is the newest version of nationally representative investigation among Chinese population aged ≥45 years. Moreover, associations between various PA dimensions (intensity, frequency, duration, and volume) and HD-associated morbidity were assessed to establish a better PA protocol for HD prevention.

## 2 Methods

### 2.1 Study design

This cross-sectional study was based on the CHARLS, which assessed the health, social and economic status of nationally representative samples covering 450 villages and 150 counties in 28 provinces (14). This survey involved Chinese community-dwelling adults aged 45 or older and their spouses. Multi-stage stratified probability-proportional-to-size sampling method was used for sample size determination. The data adopted in this study was the latest version of CHARLS (Wave 4) data available. CHARLS was approved by the Biomedical Ethics Review Committee of Peking University (Approval no. IRB00001052-11015). Written informed consents were obtained from all participants.

### 2.2 Outcome variables

Participants were asked: “Have you been diagnosed with heart attack, coronary heart disease, angina, congestive heart failure, or other heart problems by a doctor?” We considered that the individual had HD when the response was “yes”.

### 2.3 Assessment of physical activities

Respondents were asked to finish a questionnaire about weekly PA in three predefined intensity categories. Indications and examples of intensity were: (1) Vigorous physical activities (VPA): Activities that cause shortness of breath, including carrying heavy stuff, digging, hoeing, aerobic workout, bicycling at a fast speed, and riding a cargo bike/motorcycle among others; (2) Moderate physical activities (MPA): Activities that make one breath faster than usual, including carrying light stuff, bicycling at a normal speed, mopping, Tai-Chi, and speed walking; (3) Light physical activities (LPA) such as walking at home and work. Each intensity category of PA was asked to choose the frequency (times per week) and how much time of PA was conducted in one day (<10 min, 10–30 min, 30 min–2 h, 2–4 h, ≥4 h). The median daily PA time was used to calculate PA volume. The highest category for daily PA time was open-ended, then the width of the interval was obtained by referring to other intervals.

Metabolic equivalent of task (MET) was cited to calculate the volume of PA with considerations of intensity. Resting energy expenditure during quiet sitting was defined as one MET. According to the Physical Activity Guidelines Advisory Committee Scientific Report of 2018 (15), we assigned 6.0 METs, 3.1 METs, and 1.6 METs for VPA, MPA, and LPA, respectively. The weekly PA volume for each intensity category equals the product of PA frequency, the daily duration of PA, and the value assigned for each category. The total volume of PA (TPA) equals the sum of VPA, MPA, and LPA. The TPA volume was classified by quartiles.

### 2.4 Covariates

**Figure 1:**
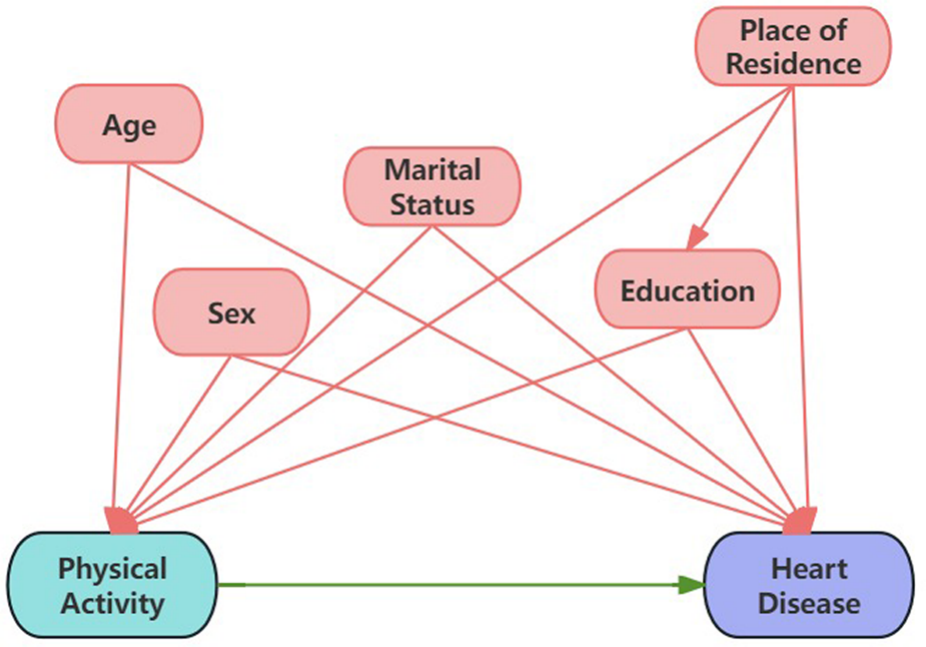
Directed acyclic graph (DAG) illustrating the causal effect of physical activity on heart disease.

Demographic characteristics, including age, sex (male/female), marital status (married/separated/divorced/widowed, and never married), education level (below junior high school, senior high school and vocational school, college and above), and place of residence (rural, urban) were self-reported. Health-related behaviors included smoking status (never smoked, quit smoking, current smoker), drinking frequency in the past (never, ≤1/month, >1/month), and sleep duration (≤7 h, 7–8 h, ≥8 h). Possible confounding variables and effect modifiers were identified through a review of the literature and integrated into a directed acyclic graph that was used to guide the modeling strategy (16–22) (Figure 1). These covariates included age, sex, marital status, education and residency. Considering potential reverse causation, smoking status, drinking frequency, and sleep duration were not included as confounders in the adjusted models.

### 2.5 Statistical analysis

STATA 16.0 was used for statistical analyses. Descriptive statistics for participant characteristics are presented according to the self-reported doctor diagnosis of HD. Continuous data are expressed as mean ± SD and analyzed with student's *t*-test. Categorical variables were expressed as counts and percentages and were compared using the chi-square test. Multiple logistic regression analysis was used to explore the association between various dimensions of PA and the risk of HD. Model building started with univariate analyses. Multivariate Model was adjusted for potential confounding factors (including age, sex, marital status, education and residency). Odds ratios (ORs) and 95% confidence intervals (CIs) were calculated to show the associations between PA and HD-associated morbidity. A restricted cubic spline (RCS) was used to assess the dose-response relationship between PA and HD risk with three knots at the 25th, 50th and 75th percentiles of PA levels. The RCS models were adjusted for age, sex, marital status, education and residency. The dose-response relationships between PA and HD for different ages and sex were also determined. Multiple imputation was used to impute missing data, and sensitivity analysis was performed on the imputed data. All tests were two-tailed, and *p* < 0.05 was considered significant.

## 3 Results

### 3.1 Demographic characteristics

Baseline parameters for demographics, lifestyle behaviors, and living conditions of cases and controls are shown in Table 1. Based on inclusion criteria, 16,927 participants from CHARLS 2018 were included in this study, of whom 8,267 (48.84%) were male, and 1,350 (7.98%) were HD patients. Age, sex, marital status, drinking frequency, smoking status, residency, education, and sleep duration were associated with HD risk.

**Table 1:** Participants’ characteristics stratified by HD.

Characteristic	Total	Heart disease	Normal	*P*-value
	(*N* = 16,927)	(*N* = 1,350)	(*N* = 15,577)	
Age, year, mean (SD)	61.17 (10.27)	60.96 (10.26)	63.59 (9.99)	0.001
Sex, *n* (%)				0.001
Male	8,267	7,700 (45.49%)	567 (3.35%)	
Female	8,660	7,877 (46.54%)	783 (4.63%)	
Marital status, *n* (%)				0.022
Married or cohabiting	14,577	13,444 (79.42%)	1,133 (6.69%)	
Separated, divorced or widowed	2,247	2,036 (12.03%)	211 (1.25%)	
Never married	103	97 (0.57%)	6 (0.04%)	
Drinking frequency, *n* (%)				0.001
≤1/month	4,656	4,381 (25.8%8)	275 (1.62%)	
>1/month	1,300	1,199 (7.08%)	101 (0.6%)	
Never	10,971	9,997 (59.06%)	974 (5.75%)	
Smoking status, *n* (%)				0.001
Current smoker	4,732	4,438 (26.22%)	294 (1.74%)	
Quit	2,318	2,089 (12.34%)	229 (1.35%)	
Never	9,877	9,050 (53.46%)	827 (4.89%)	
Residency, *n* (%)				0.001
Urban	4,641	4,188 (24.74%)	453 (2.68%)	
Rural	12,286	11,389 (67.28%)	897 (5.3%)	
Education, *n* (%)				0.051
Junior high school or less	14,778	13,627 (80.5%)	1,151 (6.8%)	
Senior high school and vocational school	2,009	1,825 (10.78%)	184 (1.09%)	
College or higher	140	125 (0.74%)	15 (0.09%)	
Sleep duration, hours, *n* (%)				0.001
<7	4,732	4,438 (26.22%)	294 (1.74%)	
7–8	2,318	2,089 (12.34%)	229 (1.35%)	
≥8	9,877	9,050 (53.46%)	827 (4.89%)	

### 3.2 Multiple logistic regression analysis of PA and HD

#### 3.2.1 Volume of PA

Multivariate logistic regression results for associations between TPA and HD are presented in Table 2. In univariate model, compared to the first quartile of TPA, a low onset risk of HD was significantly associated with a high TPA in the 3rd and 4th quartiles of TPA. After adjustment by age, sex, marital status, education, and residency, multivariate model showed the same trend of association between TPA and HD risk. Compared to the least active individuals, the risk of HD in most active participants was lower by 23% (OR 0.77 95% CI 0.65–0.92). In addition, an inverse association was detected between TPA and HD risk (*p*-trend <0.001) in both two models.

**Table 2:** Logistic regression analyses of associations between TPA and HD status.

Total PA score	Univariate model		Multivariate model	
	OR (95% CI)	*P* value	OR (95% CI)	*P* value
Q1	1		1	
Q2	0.87 (0.75, 1.01)	0.074	0.89 (0.77, 1.04)	0.143
Q3	0.75 (0.63, 0.88)	0.001	0.82 (0.69, 0.97)	0.019
Q4	0.62 (0.52, 0.73)	0.001	0.77 (0.65, 0.92)	0.004
*P*-trend	0.001		0.001	

OR, odds ratio; CI, confidence interval. Univariate Model was unadjusted; multivariate model was adjusted for age, sex, marital status, education and place of residence.

#### 3.2.2 Frequency of PA

The onset risk of HD was analyzed by the frequency of each intensity category. Compared to participants with no activity, a higher frequency of VPA was associated with a lower risk of HD, as shown in univariate model (Table 3). However, after adjusted by confounding factors, participants who performed VPA for 3–5 days per week had no significant difference in HD risk, relative to those of no activity (Table 3). After adjusting for potential confounding factors, multivariate model demonstrated a 30% lower risk of HD (OR 0.70, 95% CI 0.52–0.94) for individuals engaging in 1–2 days of VPA per week. Similarly, participants engaging in 6–7 days of VPA per week exhibited a 28% lower risk of HD (OR 0.72, 95% CI 0.61–0.85). Before adjustment, a frequency of 6–7 days MPA per week was associated with a lower risk of HD (OR 0.88, 95% CI 0.77–0.99), but it was not significant after adjustment. A higher frequency of LPA was not associated with decreased HD risk (Table 3).

**Table 3:** Associations between HD risk and PA frequency, duration, and intensity.

Variables	Univariate model		Multivariate model	
	OR (95% CI)	*P* value	OR (95% CI)	*P* value
Frequency				
VPA				
No activity	1		1	
1–2 day/week	0.61 (0.45, 0.81)	0.001	0.70 (0.52, 0.94)	0.017
3–5 day/week	0.72 (0.58, 0.90)	0.004	0.87 (0.69, 1.09)	0.223
6–7 day/week	0.59 (0.50, 0.69)	0.001	0.72 (0.61, 0.85)	0.001
MPA				
No activity	1		1	
1–2 day/week	0.88 (0.69, 1.11)	0.271	0.92 (0.73, 1.17)	0.487
3–5 day/week	0.84 (0.69, 1.02)	0.085	0.90 (0.74, 1.10)	0.316
6–7 day/week	0.88 (0.77, 0.99)	0.041	0.91 (0.80, 1.04)	0.163
LPA				
No activity	1		1	
1–2 day/week	0.72 (0.51, 1.04)	0.077	0.74 (0.51, 1.06)	0.097
3–5 day/week	0.89 (0.70, 1.13)	0.336	0.92 (0.72, 1.18)	0.516
6–7 day/week	0.96 (0.83, 1.11)	0.556	0.99 (0.85, 1.15)	0.860
Duration				
VPA				
Inactive	1		1	
10–29 min/day	0.49 (0.24, 1.00)	0.050	0.55 (0.27, 1.13)	0.106
30–119 min/day	0.72 (0.56, 0.92)	0.010	0.80 (0.62, 1.04)	0.093
≥120 min/day	0.61 (0.52, 0.70)	0.001	0.75 (0.64, 0.87)	0.001
MPA				
Inactive	1		1	
10–29 min/day	0.88 (0.68, 1.12)	0.291	0.87 (0.68, 1.11)	0.260
30–119 min/day	0.95 (0.82, 1.10)	0.504	0.96 (0.82, 1.11)	0.559
≥120 min/day	0.80 (0.69, 0.92)	0.002	0.88 (0.76, 1.02)	0.096
LPA				
Inactive	1		1	
10–29 min/day	0.93 (0.74, 1.17)	0.536	0.95 (0.75, 1.19)	0.627
30–119 min/day	1.00 (0.85, 1.17)	0.972	1.00 (0.85, 1.17)	0.984
≥120 min/day	0.87 (0.74, 1.03)	0.104	0.94 (0.79, 1.11)	0.473

VPA, vigorous physical activity; MPA, moderate physical activity; LPA, light physical activity; OR, odds ratio; CI, confidence interval. Univariate model was unadjusted; multivariate model was adjusted for age, sex, marital status, education and place of residence.

#### 3.2.3 Duration of physical activity

The logistic regression results for the duration of different intensity PA and the risk of HD were presented in Table 3. In univariate model, a longer duration of VPA was associated with a lower risk of HD compared to inactive participants. Participants engaging in VPA for more than 30 min per day had a lower risk of HD (*p* < 0.05), as did those engaging in MPA for more than 120 min per day (*p* < 0.05). However, there was no difference in the risk of HD among different durations of LPA. After adjusting for multiple variables in multivariate model, participants engaging in VPA for more than 120 min per day had a lower risk of HD (*p* < 0.05). There was no significant association found between the duration of MPA and LPA and the risk of HD (*p* > 0.05).

#### 3.2.4 Dose-response relationship between PA and HD risk

**Figure 2:**
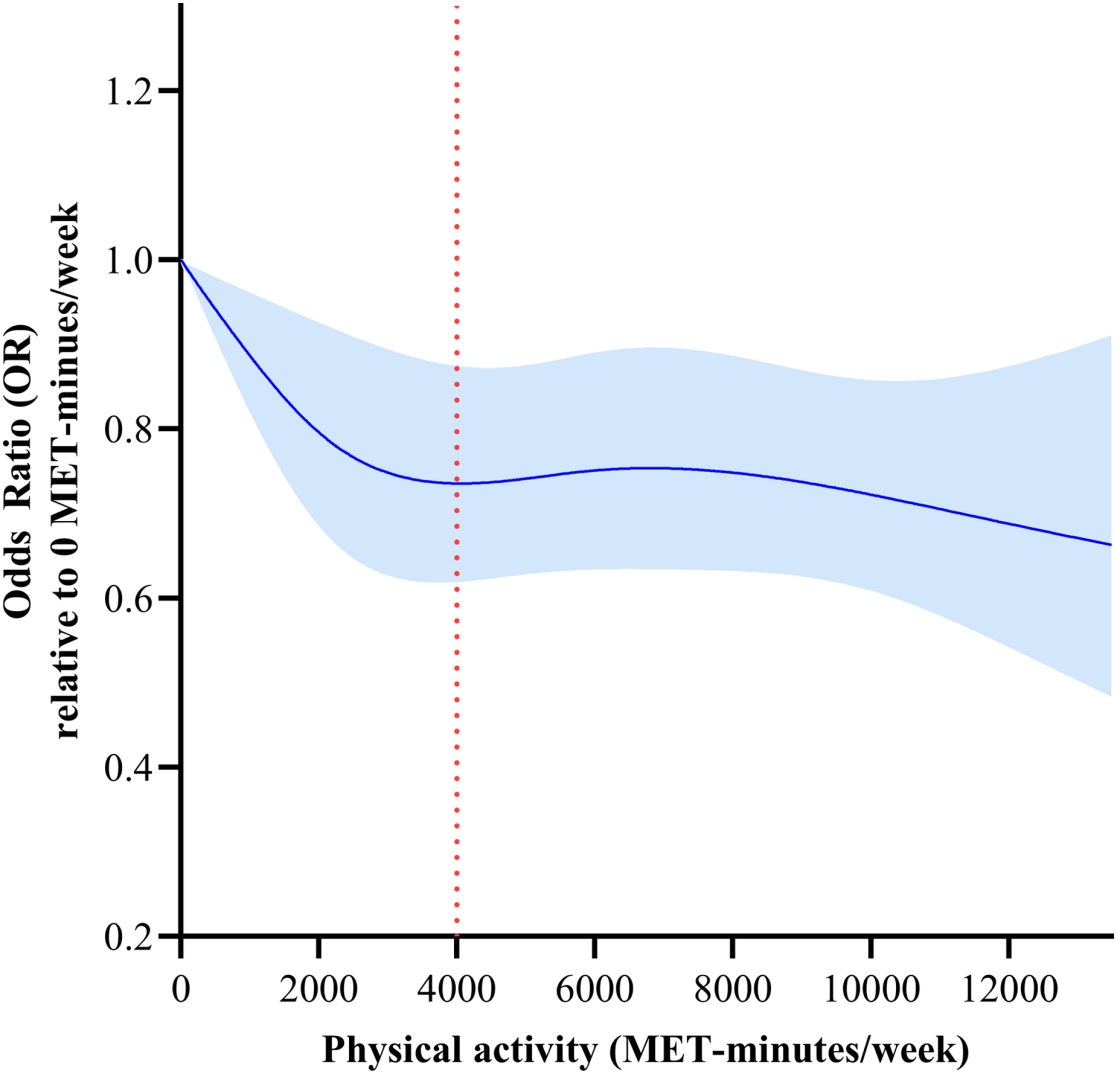
Dose-response relationships between total physical activity (METs-min/week) and HD risk. Models were adjusted for age, sex, marital status, education, residency, smoking status, drinking frequency and sleep duration. This graph shows ORs (solid line) with 95% CI (shaded area).

The dose-response relationship between TPA and HD risk is shown in Figure 2. A nonlinear dose-response relationship with steeper reductions in HD risk at low TPA levels than at high TPA levels was detected (*p* overall < 0.001, *p* for non-linearity = 0.014). The risk of HD decreased rapidly as TPA level increased from 0 to 4,000 METs-min/week, but more slowly for those with TPA above 4,000 METs-min/week. The risk of HD was 26% lower in participants with TPA of 4,000 METs-min/week than in those with no physical activity (OR 0.74, 95% CI 0.62–0.89).

#### 3.2.5 Interaction and subgroup analysis

**Figure 3:**
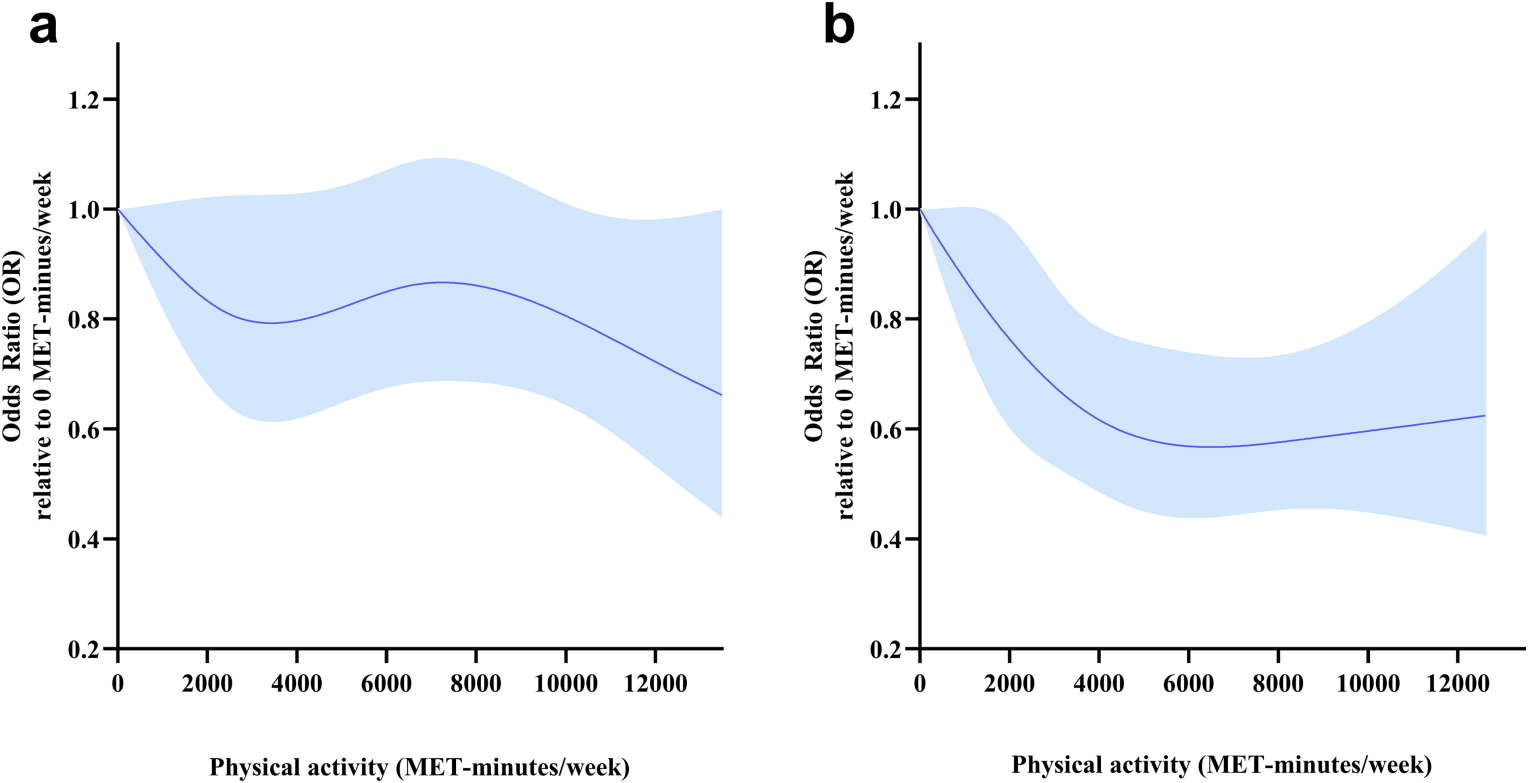
Dose-response relationships between total physical activity (METs-min/week) and HD risk for different age groups. Models were adjusted for sex, marital status, education, residency, smoking status, drinking frequency and sleep duration. This graph shows ORs (solid line) with 95% CI (Shaded area). **(a)** Participants aged <65 years. **(b)** Participants aged ≥65 years.

To interpret the potential heterogenous effect in people with different demographic characteristics, we ran the statistical models to examine the interaction of age and physical activity. There was a significant interaction between age and PA variability on HD (*p* for interaction = 0.049). Age-stratified analysis was conducted to interpret associations among participants with different ages. The risk of HD decreased with increasing TPA. A reverse linear dose-response relationship was found between TPA and HD for participants aged <65 years (*p* for non-linearity >0.05, Figure 3a), while a nonlinear dose-response relationship between TPA and HD was observed in participants aged ≥65 years (*p* for non-linearity < 0.05, Figure 3b). Among participants aged ≥65 years, a 36% lower risk of HD was associated with a TPA level of about 4,000 METs-min/week (OR 0.64, 95% CI 0.50–0.81).

**Figure 4:**
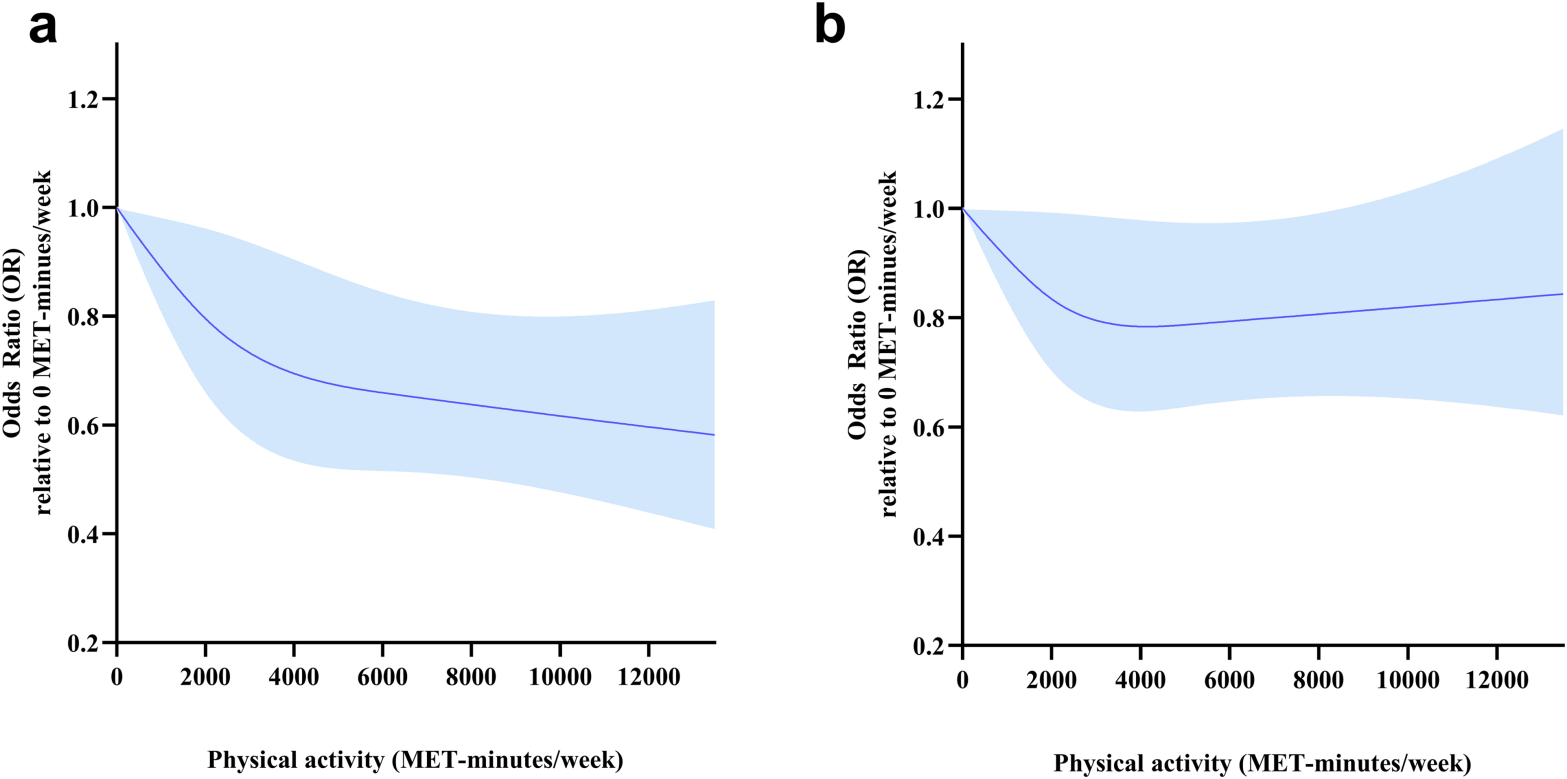
Dose-response relationships between total physical activity (METs-min/week) and HD risk for different sex groups. Models were adjusted for age, marital status, education, residency, smoking status, drinking frequency and sleep duration. This graph shows ORs (solid line) with 95% CI (shaded area). **(a)** Males. **(b)** Females.

There was no significant interaction between sex and physical activity variability on HD (*p* for interaction = 0.234). Given the observed sex-specific variability in HD as indicated by the results of the chi-square test, further subgroup analyses were conducted to elucidate the potential dose-response relationship between PA and HD within the context of sex. In the sex-stratified analysis (Figure 4), there was a linear dose-response relationship between TPA and HD risk in males (p_non-linearity_ > 0.05), and the OR for HD decreased sharply as the PA increased. However, the dose-response relationship between TPA and HD risk was not statistically significant in women.

**Figure 5:**
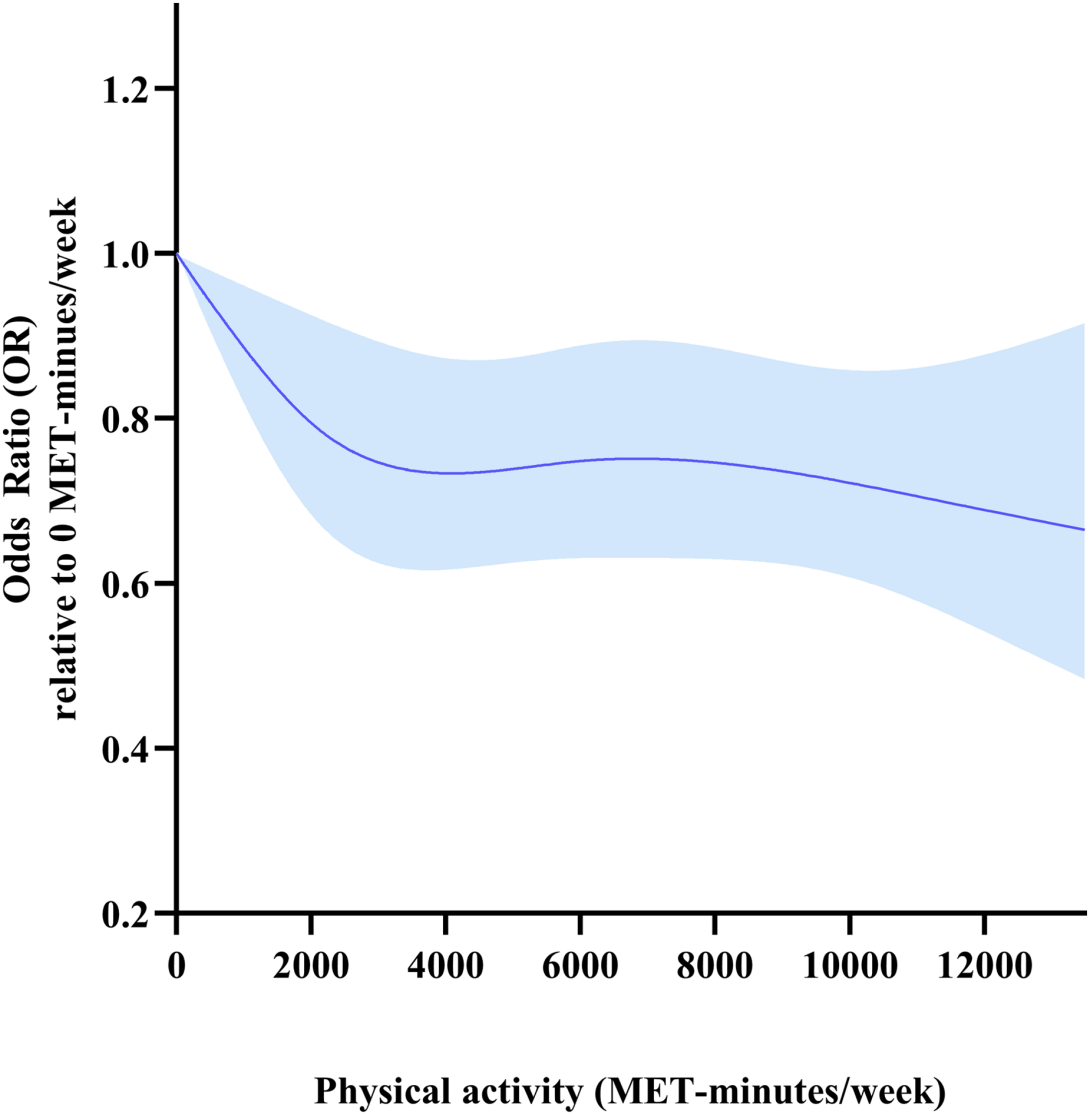
Sensitivity analyses of dose-response relationships between total physical activity (METs·min/week) and risk of heart diseases. Models were adjusted for age, sex, marital status, education, residency, smoking status, drinking frequency and sleep duration. This graph shows ORs (solid line) with 95% CI (shaded area).

Missing values were imputed using multiple imputation, 19,498 participants were included in the sensitivity analysis. A similar nonlinear dose-response relationship between TPA and HD risk was observed in the sensitivity analysis (*p*_overall_ < 0.001, *p*_non-linearity_ = 0.025), and the shape of the dose-response curve was similar to the main analysis (Figure 5).

## 4 Discussion

We established that a higher TPA was associated with a low HD risk, and an inverse non-linear dose-response relationship was detected. The plateau of TPA was 4,000 METs-min/week, with a 26% reduction in HD risk. A higher frequency and longer duration of VPA were associated with a low HD risk, while the association was insignificant in both MPA and LPA after adjustment of demographics, lifestyle behaviors, and living conditions. Subgroup analysis revealed an inverse nonlinear dose-response relationship between TPA and HD for participants aged ≥65 years, and a 36% lower risk of HD was associated with a TPA level of 4,000 METs-min/week in participants aged ≥65 years. A linear dose-response relationship between TPA and HD risk was detected in males instead of females.

TPA was inversely associated with HD risk. Insufficient PA increases the risk for cardiovascular diseases, especially coronary arterial disease, and myocardial infarction (23). Exercise training should be part of cardiac rehabilitation programs. Cattadori et al. (24) reported that PA protects against the onset of HF and provides secondary prevention for HF patients. Moreover, exercise impairment is a prognostic factor for HF (24). PA is recommended for congenital heart disease patients (25). We found that the higher the TPA levels, the lower the HD risk, thus we postulated that a lack of PA is a risk factor for HD, and TPA volume is a prospective indicator for HD risk.

The intensity of PA is a vital indicator for preventing HD risk. Previous studies reported an inverse association between relative intensities of physical activity (an individual's perceived level of exertion) and coronary heart disease risk, and moderate-intensity physical activity was recommended to reduce coronary heart disease risks (26). Regular vigorous exercises also reduce the risk of developing cardiovascular diseases, compared to moderate-intensity activity (27). Lee et al. (28) reported an inverse association between PA and coronary heart disease-associated morbidity while light-to-moderate activity was associated with decreased coronary heart disease morbidity among women. Sesso et al. (29) found a nonsignificant inverse association between PA and coronary heart disease occurrence. We established that VPA is correlated with a lower HD risk, even when performed at a low frequency or for a short duration. Importantly, compared to participants without physical activity, vigorous-intensity activity was associated with reduced risk of independent of the volume of TPA, which is in accordance with the conclusion that sedentary people can benefit from regular short activity periods of as little as 1 min (30). Higher intensity activities provide greater health benefits with a dose-response relationship (31). Although light intensity PA can confer accumulating health benefits (32), and moderate-intensity PA is recommended in several physical activity guidelines (33, 34), our findings indicate that the benefits of accumulating light-moderate intensity PA for reducing the risk of HD may be dismissed by other covariates. Thus, vigorous-intensity PA is strongly recommended to reduce the risk of HD. The recommendation of intensity for HD patients has not been fully confirmed. Light to moderate intensity PA has been suggested in some guidelines (35), but, habitual vigorous activities are not associated with increased risk of subsequent myocardial infarctions in coronary heart disease patients (36). Patients with specific lesions or complications may require counseling regarding precautions and recommendations.

There was a nonlinear dose-response relationship between HD risk and TPA levels. Low TPA levels were associated with steeper reductions in HD risk, and the percentage of risk reduction was less with the increase in TPA. According to physical activity guidelines for Americans, benefits begin with a moderate PA volume, but greater amounts of PA result in further reductions in cardiovascular disease risks (37). This theory was in accordance with our results. A plateau was reached at 4,000 METs-min/week. Assuming causality, PA levels should be increased to reduce HD risk. There is an economical level of TPA that is necessary to reduce the risk of HD, rather than “the more, the better”. An economical level of TPA to reduce the risk of HD by 36% for participants aged than 65 years was established to be 4,000 METs-min/week, which is higher than some physical activity guidelines (33, 34), it would need a higher TPA to prevent instead of improving HD (37). Interestingly, the risk reduction of HD was more sensitive in males than females. Consistently, the strength of association between daily step count and CVD risk appeared to be stronger in men than in women (38). Sex difference in the recommendation of PA also exists, and it was encouraged to perform a higher TPA for women compared to men to prevent HD. Overall, higher TPA levels are recommended to prevent HD risks, and a basic TPA score was recommended for the best economic protection effect.

This study has some strengths, including the use of a nationwide representative sample covering 28 provinces in mainland China and the use of the CHARLS database, which has proven its validity and reliability in a large number of studies (14). This study also has some limitations. First, causal association between PA and the risk of HD cannot be interpreted by a cross-sectional study. Second, recall bias is not avoidable in self-reported questionnaires, and the species of HD were not distinguished in the questionnaire. Third, leisure-time PA and work-related PA were mixed so that the PA paradox may be neglected (39). Finally, the PA data were collected using self-reported questionnaires, which means that PA was not strictly continuous when categorized into different intensities. The various intensities of physical activity were somewhat isolated from each other, and sedentary behavior was not accounted for in the analysis, which restricted our ability to analyze one domain of PA intensity while adjusting for the effects of other intensities based on 24-h activities. This limitation may introduce bias when interpreting the differential effects of various intensities. In future studies, more consideration should be given to using accelerometers to obtain physical activity data, comprehensively considering physical activity, sedentary behavior and sleep, and conducting more in-depth analysis based on isochronous substitution and other models.

## 5 Conclusion

An inverse non-linear dose-response association was detected between total volume of physical activity and HD risk. As physical activity increased to 4,000 METs-min/week, HD risk in the overall population decreased by approximately 26%, while further increases in physical activity did not produce any further marked reduction in the risk. A vigorous intensity of physical activity was associated with a reduced risk of HD and is strongly recommended.

## Data Availability

The original contributions presented in the study are included in the article/Supplementary Material, further inquiries can be directed to the corresponding author.
